# Immune-related genes of the larval *Holotrichia parallela* in response to entomopathogenic nematodes *Heterorhabditis beicherriana* LF

**DOI:** 10.1186/s12864-021-07506-4

**Published:** 2021-03-17

**Authors:** Ertao Li, Jianhui Qin, Honglin Feng, Jinqiao Li, Xiaofeng Li, Innocent Nyamwasa, Yazhong Cao, Weibin Ruan, Kebin Li, Jiao Yin

**Affiliations:** 1 State Key Laboratory for Biology of Plant Diseases and Insect Pests, Institute of Plant Protection, Chinese Academy of Agricultural Sciences, Yuanmingyuan West Road, Beijing, 100193 China; 2 Boyce Thompson Institute for Plant Research, Cornell University, 533 Tower Road, Ithaca, NY 14853 USA; 3 College of Life Sciences, Nankai University, Tianjin, 300071 P.R. China

**Keywords:** Entomopathogenic nematode, *Holotrichia parallela*, Immune response

## Abstract

**Background:**

Entomopathogenic nematodes (EPNs) emerge as compatible alternatives to conventional insecticides in controlling *Holotrichia parallela* larvae (Coleoptera: Scarabaeidae). However, the immune responses of *H. parallela* against EPNs infection remain unclear.

**Results:**

In present research, RNA-Seq was firstly performed. A total of 89,427 and 85,741 unigenes were achieved from the midgut of *H. parallela* larvae treated with *Heterorhabditis beicherriana* LF for 24 and 72 h, respectively; 2545 and 3156 unigenes were differentially regulated, respectively. Among those differentially expressed genes (DEGs), 74 were identified potentially related to the immune response. Notably, some immune-related genes, such as peptidoglycan recognition protein SC1 (PGRP-SC1), pro-phenoloxidase activating enzyme-I (PPAE-I) and glutathione s-transferase (GST), were induced at both treatment points. Bioinformatics analysis showed that PGRP-SC1, PPAE-I and GST were all involved in anti-parasitic immune process. Quantitative real-time PCR (qRT-PCR) results showed that the three immune-related genes were expressed in all developmental stages; PGRP-SC1 and PPAE-I had higher expressions in midgut and fat body, respectively, while GST exhibited high expression in both of them. Moreover, in vivo silencing of them resulted in increased susceptibility of *H. parallela* larvae to *H. beicherriana* LF.

**Conclusion:**

These results suggest that *H. parallela* PGRP-SC1, PPAE-I and GST are involved in the immune responses to resist *H. beicherriana* LF infection. This study provides the first comprehensive transcriptome resource of *H. parallela* exposure to nematode challenge that will help to support further comparative studies on host-EPN interactions.

**Supplementary Information:**

The online version contains supplementary material available at 10.1186/s12864-021-07506-4.

## Background

The dark black chafer (*Holotrichia parallela* Motschulsky) is a polyphagous pest that impacts many major crops, pastures and herbs in East Asia, particularly in China [1–3]. The larvae, white grubs, live in soil and prefer to feed on plant roots, causing an average economic loss of more than 15% per year [4]; in serious cases, the losses may exceed 50% [5]. In recent years, the implementation of agricultural measures, such as no-tillage and shallow tillage systems, straw return, has created unique conditions for the survival and quantity of white grubs [6, 7]. The application of chemical insecticides has been widely used as an effective measure in white grub management but resulted in soil and groundwater pollution [8, 9].

Entomopathogenic nematodes (EPNs), including Steinernematidae and Heterorhabditidae, are regarded as potential biological control agents to control a wide range of insect pests, especially those that occur in soil [10]. Similar to predators, the infective juveniles (IJs) of EPNs possess chemoreceptors that can actively search for susceptible hosts living in the soil [11, 12]. Once IJs have captured susceptible hosts, they will enter the host through their natural body openings such as stoma, valve and anus, eventually reaching the host’s haemocoel and gut region, and then the IJs release the symbiotic bacteria that secrete proteases, protoxins and other insecticidal substances complex to establish infection [13]. More importantly, EPNs are highly virulent, killing the pest quickly, meanwhile they are safe to vertebrates and plants [14]. In 1929, EPNs were first discovered as the parasites of Japanese beetle (*Popillia japonica*) in New Jersey [15] and produced a high mortality to white grubs [16]. To date, approximately seven EPN species, including *Steinernema scarabaei*, *Steinernema glaseri*, *Heterorhabditis bacteriophora* H06, *Heterorhabditis* spp., *Steinernema longicaudum* X-7, *Heterorhabditis indica* LN2 and *Heterorhabditis beicherriana*, have shown potential control abilities for *P. japonica*, masked chafers (*Cyclocephala borealis* Arrow), *Holotrichia oblita* and *H. parallela* [7, 17–21]. However, the pathogenicity of nematodes is unstable in practical applications, and the control effect is unsatisfactory [22, 23]. The deadliest cause was that white grubs possess a strong immune system [24], which may effectively resist nematodes to establish infection [25]. Specifically, white grubs can recognize EPNs through the sensitive immune system and can encapsulate or melanize invading nematodes [26–28].

Generally, the immune system of insects will be activated after infection by parasites or pathogens. First, pattern recognition proteins (PRPs) primarily recognize and tag invaders [29–32]. Then, PRPs rely on the cascade reaction of serine proteases (SPs) to continuously expand signals and eventually transmit the signals to the nucleus [33]. Finally, antimicrobial peptides (AMPs) were used to perform phagocytosis, nodulation and encapsulation depending on the invader [34, 35], and other immune-related genes, including GSTs, heat shock protein (HSPs) and superoxide dismutase (SOD), were also involved in the anti-parasitic immune response [24].

Recently, mRNA sequencing (RNA-Seq or transcriptome sequencing) has been widely applied to identify immune-related genes and study the molecular basis of host-bacterial and/or host-parasite interactions. Moreover, this method also provides comprehensive insight into the immune gene repertoire of non-model insects, and this technology has become a reliable method to identify and analyze differentially expressed genes (DEGs) as targets for pest control [36, 37]. For instance, transcriptome sequencing results of *Drosophila* response to *H. bacteriophora* H222 infection indicated that most of the strongly induced genes were implicated in immune responses [38]; Many immune-related genes (such as PRPs, immune-related signal transduction proteins, AMPs and cellular response proteins) were induced in the white-spotted flower chafer (*Protaetia brevitarsis seulensis* Kolbe) with *Escherichia coli* and *Saccharomyces cerevisiae* challenge [24]; *Heliothis virescens* response to *H. bacteriophora* infection results indicated that insect immune response genes were induced upon nematode invasion, but the majority of these genes were suppressed after the release of symbiotic bacteria by the nematode [39]. However, the molecular immune mechanism of *H. parallela* against EPNs infection has not been elucidated.

In this study, we primarily generated the first comprehensive transcriptome resource regarding *H. parallela* larvae in response to the nematode infection and screened the DEGs associated with immune defence. Then, we predicted the roles of three key immune-related genes in anti-parasite immune processes based on bioinformatics analysis. After that, we performed qRT-PCR to investigate the spatiotemporal expression patterns of these key immune-related genes. Finally, we silenced these immune-related genes to test their effects on the susceptibility of *H. parallela* larvae to *H. beicherriana* LF.

## Results

### Transcriptome overview of *Holotrichia parallela* midgut

Through sequencing, a total of 89,427 and 85,741 unigenes were generated from the midgut samples of *H. parallela* larvae treated with the nematode *H. beicherriana* LF for 24 and 72 h, respectively. The specific statistics of transcriptomes are shown in Table 1.

**Table 1:** Summary statistics of transcriptomes

Summary	Hp-CK1	Hp-LF1	Hp-CK3	Hp-LF3
Raw reads number	47,126,795	45,510,292	45,690,800	46,603,740
Raw bases number	7,049,508,850	6,826,543,800	6,816,176,000	7,018,329,600
Clean reads number	46,156,622	44,287,283	44,894,961	45,457,525
Clean bases number	6,885,817,500	6,643,092,450	6,847,377,850	6,818,628,750
Clean Q30 bases rate (%)	95.13	94.92	95.58	95.50
Percen GC of trinity/ unigenes (%)	35.77/35.28	35.98/35.45	35.47/35.34	35.85/35.42
Count of trinity/ unigenes	156,263/70976	175,496/89427	167,584/79571	176,586/85741
Mean length of trinity/ unigenes	890.87/771.14	928.46/756.53	945.18/799.56	899.75/786.42
N50 of trinity/ unigenes	1542/1200	1598/1495	1613/1299	1687/1428

Note: Hp: *Holotrichia parallela*; LF1: postexposure to *H. beicherriana* LF for 24 h; LF3: postexposure to *H. beicherriana* LF for 72 h. The data are presented as two biological replicates. Clean Q30 bases rate is identified as the proportion of bases with a mass value greater than 30 (error rate less than 0.1%) in the total sequence after filtration. N50 is identified as the sequence length of the shorted contig at 50% of the total genome length

**Fig. 1:**
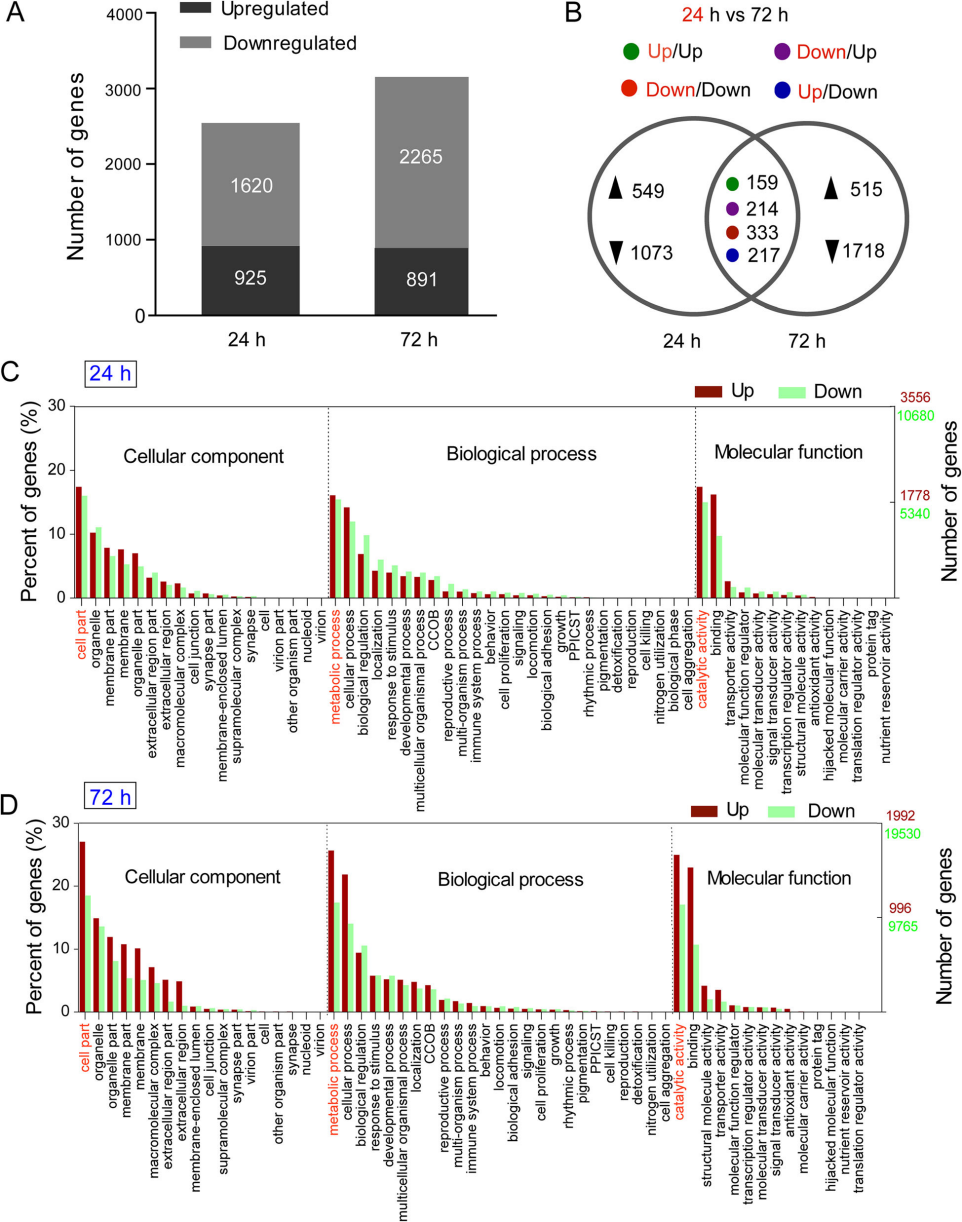
Transcriptome overview of *Holotrichia parallela* larvae after treatment with *Heterorhabditis beicherriana* LF. **a** The number of genes differentially expressed in *H. parallela* after treatment with nematodes for 24 and 72 h, respectively. **b** Venn diagrams showing the number of *H. parallela* genes that are differentially expressed (upregulated or downregulated) at 24 h only or at 72 h only or at both time-points after treatment with nematodes. Expression patterns are indicated (UP/UP: gene upregulation at both 24 and 72 h, DOWN/UP: gene downregulation at 24 h and upregulation at 72 h, DOWN/DOWN: gene downregulation at both time-points, UP/DOWN: gene upregulation at 24 h and downregulation at 72 h). **c** and **d** Gene Ontology classification of the *H. parallela* larvae midgut transcript with Blast2GO program after treatment with *H. beicherriana* LF for 24 and 72 h. CCOB: cellular component organization or biogenesis; PPICST: presynaptic process involved in chemical synaptic transmission

After treating the larvae of *H. parallela* with nematodes for 24 h, 2545 genes were significantly differentially expressed compared to the sterile water treatment, among which 925 were upregulated and 1620 were downregulated. At 72 h postexposing, 3156 genes were significantly differentially expressed, among which 891 were upregulated and 2265 were downregulated (Fig. 1a). In addition, we found that 159 genes were upregulated and 333 genes were downregulated at both time- poins; 214 genes were downregulated at 24 h and upregulated at 72 h; 217 genes were upregulated at 24 h and downregulated at 72 h (Fig. 1b).

Of all the unigenes, approximately 14.90% were annotated to seven databases, including NT, NR, UniProt database, RNAMMER, eggNOG, KEGG and GO. Using the GO classification (Fig. 1c-d), these DEGs participated in at least 58 biological activities and were characterized into three groups: cellular component, biological process and molecular function. For the cellular component, the high percentage of genes were concentrated in the cell part category (24 h, up: 1242 genes, 17.46%; down: 3423 genes, 16.03%. 72 h, up: 1078 genes, 27.05%; down: 7244 genes, 18.51%). The biological process group showed a significant percentage of genes assigned to metabolic process category (24 h, up: 1148 genes, 16.14%; down: 2559 genes, 11.98%. 72 h, up: 1022 genes, 25.65%; down: 5490 genes, 14.03%). For molecular function, the most represented ontology was catalytic activity (24 h, up: 1240 genes, 17.44%; down: 2077 genes, 9.73%. 72 h, up: 996 genes, 24.99%; down: 4187 genes, 10.7%).

### Immune defensive pathways

Considering that the immune response between *H. parallela* and *H. beicherriana* LF interaction is the key factor in the successful control of nematode parasitism, the transcripts related to immune defence responses upon nematode infection were selected. Through a complete search of the immune-related terms from the differential gene expression data, we identified a total of 74 DEGs that might be related to the immune defence mechanism in *H. parallela*. In this instance, differentially expressed transcripts were distributed among different immune response pathways, including recognition, activation of signalling pathways and production of effector molecules (Table 2). The results showed that most of the genes showed no significant expression change after treatment with nematodes for 24 h, and many genes showed depressed expression after treatment with nematodes for 72 h relative to the control. However, some transcripts were induced for both treatment points, such as PGRP-SC1, PPAE-I and GST, indicating that those genes may be involved in the immune responses to effectively resist nematode infection. The annotations of these genes in transcriptome data are summarized in Supplementary Table S1. These sequence data have been submitted to the GenBank databases under accession number DN15104, DN15190 and DN16733, respectively.

**Table 2:** List of transcripts associated with immune defence responses upon *Heterorhabditis beicherriana* LF infection

Unigene ID	Description	Fold Change	
		24 h	72 h
**Recognition**			
TRINITY_DN9063_c0_g1	Putative peptidoglycan binding domain	+ 2.06	+ 2.13
TRINITY_DN14716_c5_g3	Peptidoglycan recognition protein 2	=	−0.4
TRINITY_DN15104_c0_g1	Peptidoglycan recognition protein SC1a/b-like	+ 4.20	+ 2.79
TRINITY_DN18918_c0_g2	C-type lectin domain family	=	+ 5.40
TRINITY_DN10984_c0_g1	C-type lectin	−0.26	=
TRINITY_DN16803_c1_g1	C-type lectin domain family 16, member A	=	−0.19
TRINITY_DN14096_c0_g6	Tubulointerstitial nephritis antigen-like	−0.44	=
TRINITY_DN11464_c1_g3	Flocculation protein FLO11-like	=	−0.01
TRINITY_DN15762_c1_g2	Flocculation protein FLO11 isoform X3	=	−0.11
TRINITY_DN10144_c0_g1	Scavenger receptor class B, member	+ 2.44	=
TRINITY_DN20348_c0_g1	Scavenger receptor cysteine-rich domain	+ 2.14	=
TRINITY_DN10223_c0_g1	Scavenger receptor class B member 1	−0.28	+ 3.80
TRINITY_DN20348_c0_g1	Scavenger receptor activity	+ 2.14	=
TRINITY_DN10537_c0_g1	Somatomedin_B	−0.50	− 0.00
**Activation of signalling pathway**			
TRINITY_DN16123_c1_g1	Tyrosine-protein phosphatase Lar isoform X2	=	−0.04
TRINITY_DN12585_c0_g9	Receptor-type tyrosine-protein phosphatase kappa	–	+ 6.63
TRINITY_DN14569_c0_g1	Tyrosine-protein phosphatase 69D	−0.32	− 0.02
TRINITY_DN11667_c0_g2	Tyrosine-protein phosphatase non-receptor type 14	=	−0.13
TRINITY_DN20113_c2_g1	Tyrosine-protein phosphatase non-receptor type 23	=	−0.36
TRINITY_DN14547_c0_g2	Tyrosine-protein phosphatase non-receptor type 4	−0.39	−0.05
TRINITY_DN16319_c2_g1	Receptor-type tyrosine-protein phosphatase N2	=	−0.07
TRINITY_DN17938_c0_g1	Tyrosine-protein phosphatase non-receptor type 7	+ 2.05	−0.28
TRINITY_DN13499_c0_g2	Receptor-type tyrosine-protein phosphatase T	−0.18	−0.14
TRINITY_DN20215_c1_g2	Tyrosine-protein phosphatase 10D	=	−0.49
TRINITY_DN20215_c1_g4	Receptor-type tyrosine-protein phosphatase beta	=	−0.15
TRINITY_DN15387_c0_g1	Zinc finger protein 1	−0.04	−0.00
TRINITY_DN18572_c2_g2	Zinc finger protein 711-like	−0.27	−0.41
TRINITY_DN12683_c0_g1	Zinc finger protein 182	=	−0.46
TRINITY_DN15982_c3_g10	Zinc finger protein Gfi-1	=	−0.09
TRINITY_DN12684_c0_g1	Zinc finger protein 26	=	−0.09
TRINITY_DN18098_c2_g2	Zinc finger protein 710	=	−0.11
TRINITY_DN14971_c1_g1	Zinc finger protein 782	−0.31	−0.03
TRINITY_DN15174_c0_g2	Zinc finger protein 431-like isoform X2	−0.39	−0.00
TRINITY_DN14699_c1_g3	JNK_SAPK-associated protein-1	=	−0.25
TRINITY_DN7086_c0_g1	Transcription factor Sox-9-B-like	−0.24	−0.32
TRINITY_DN18891_c1_g2	Interferon-related developmental regulator 2	+ 2.06	+ 4.00
TRINITY_DN18878_c0_g2	Protein lingerer	=	−0.19
TRINITY_DN18219_c2_g1	Serine protease inhibitor 42Dd	=	−0.26
TRINITY_DN15260_c0_g2	Serine protease snake	+ 5.77	+ 4.49
TRINITY_DN18055_c3_g1	Serine protease inhibitor 88Ea	−0.06	− 0.01
TRINITY_DN15190_c0_g1	Pro-phenoloxidase activating enzyme-I precursor	+ 3.47	= (1.21)
**Production of effector molecules**			
TRINITY_DN13192_c1_g2	Chorion peroxidase-like	−0.23	−0.03
TRINITY_DN10094_c0_g1	Attacin_C	−0.15	=
TRINITY_DN13744_c1_g3	ATP^†^-dependent RNA helicase p62	=	−0.49
TRINITY_DN11082_c2_g2	ATP-dependent RNA helicase	=	−0.04
TRINITY_DN17995_c3_g1	ATP-dependent RNA helicase WM6	=	−0.14
TRINITY_DN16001_c1_g1	ATP-dependent RNA helicase DHX8	−0.48	−0.26
TRINITY_DN20149_c0_g1	ATP-dependent RNA helicase DDX5/DBP2	=	−0.17
TRINITY_DN14053_c1_g1	ATP-dependent RNA helicase Ddx1	=	−0.26
TRINITY_DN16098_c1_g1	ATP-dependent RNA helicase A	−0.38	−0.16
TRINITY_DN15118_c0_g3	ATP-dependent RNA helicase DDX24	=	−0.12
TRINITY_DN19567_c0_g2	ATP-dependent RNA helicase TDRD12	−0.26	−0.06
TRINITY_DN14329_c0_g2	Spermatogenesis-associated protein 20	=	−0.28
TRINITY_DN18323_c2_g1	Spermatogenesis-associated protein 13	−0.29	−0.09
TRINITY_DN17965_c1_g1	Spermatogenesis-associated protein 5	=	−0.28
TRINITY_DN18625_c3_g1	Spermatogenesis-associated protein 13-like isoform X1	=	−0.25
TRINITY_DN14329_c0_g2	Spermatogenesis-associated protein 2	=	−0.28
TRINITY_DN11114_c0_g1	Probable chitinase 10	=	−0.08
TRINITY_DN9400_c0_g1	Probable chitinase 2	=	−0.20
TRINITY_DN11052_c6_g2	Protein takeout-like	−0.14	−0.49
TRINITY_DN17266_c1_g3	Gamma-glutamyltransferase activity	−0.13	−0.21
TRINITY_DN14465_c2_g4	Hemocyte protein-glutamine gamma-glutamyltransferase	−0.16	−0.26
TRINITY_DN16733_c0_g1	Glutathione-S-transferase	+ 4.46	+ 3.61
TRINITY_DN10980_c0_g1	Phenoloxidase subunit 1	−0.23	−0.25
TRINITY_DN15456_c3_g3	Nitric oxide-associated protein 1	=	−0.22
TRINITY_DN16202_c0_g1	Nitric oxide synthase interacting protein	=	−0.63
TRINITY_DN15462_c1_g1	Heat shock protein	+ 4.89	−0.39
TRINITY_DN9876_c0_g1	Heat shock protein 26	=	+ 4.53
TRINITY_DN6424_c0_g1	Heat shock protein 90	−0.34	=
TRINITY_DN8346_c0_g1	Heat shock protein TC005094	−0.24	=
TRINITY_DN16097_c1_g1	Heat shock protein 75 kDa	=	−0.33
TRINITY_DN19512_c2_g3	Heat shock protein 67B2	+ 14.52	=
TRINITY_DN11437_c3_g4	Farnesyl pyrophosphate synthase	=	−0.09
TRINITY_DN13560_c2_g5	Protein farnesyltransferase	=	−0.08

Note: Symbols +, − and = indicate significant upregulation, downregulation and no significant expression change, respectively. †: Adenosine triphosphate

### Bioinformatics analysis of PGRP-SC1, PPAE-I and GST

**Fig. 2:**
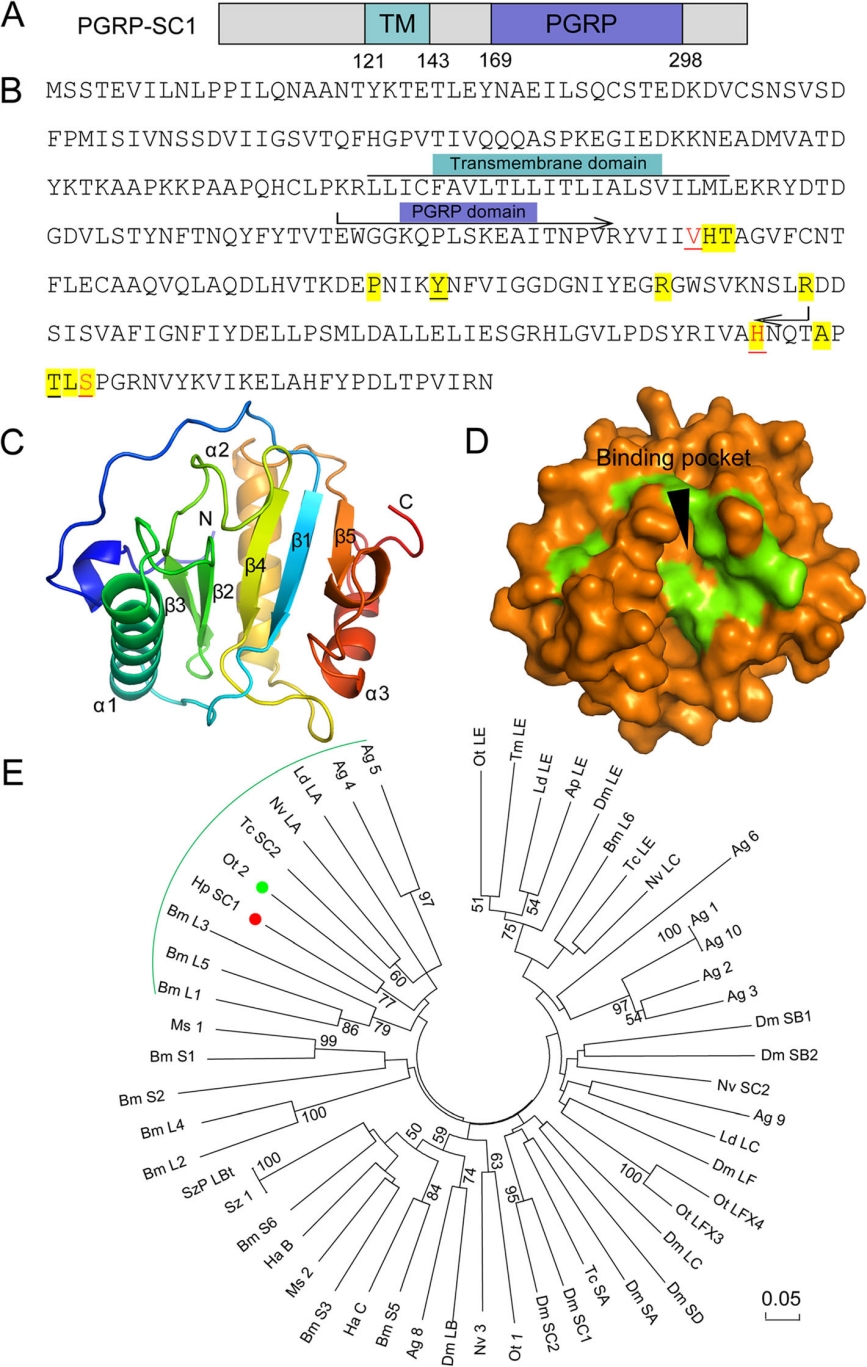
Bioinformatics analysis of *Holotrichia parallela* PGRP-SC1. **a** Schematic representation of *H. parallela* PGRP-SC1. The transmembrane domain (TM) and PGRP homologous domain (PGRP) are indicated in wathet blue and purple boxes, respectively. **b** Sequence analysis of *H. parallela* PGRP-SC1. The domain division (TM and PGRP) are indicated above the sequences. The amidase catalytic site is underlined. The Zn binding residues are shown in red. The substrate binding site are shown in yellow shading. **c** The overall structure of *H. parallela* PGRP-SC1. The N-terminus (N), C-terminus (C), *α*-helices and *β*-strands are labeled. **d** Surface of *H. parallela* PGRP-SC1 structure. Substrate binding sites are colored green. **e** Phylogenetic analysis of *H. parallela* PGRP-SC1. The red and green circles highlight *H. parallela* PGRP-SC1 and the closely homologous specie PGRP, respectively. Numbers at the nodes were bootstrap values as percentage and only bootstrap values greater than 50 are shown. The same for Figs. 3-e and 4-e. Scale bar, 0.05 substitutions per site

Based on the sequence analysis, PGRP-SC1 lacked a signal peptide but contained a transmembrane segment, which indicated that it may function on the cell membrane to recognize and bind pathogens. Moreover, PGRP-SC1 contains a highly conserved homologous PGRP domain consisting of approximately 130 amino acid residues at the shed terminal (Fig. 2a). It has also been noticed that two of five key residues responsible for zinc binding and amidase catalytic activity (His^18^, Tyr^47^, His^123^, Lys^129^ (Thr in *D. melanogaster* PGRP-LB/SC1/SC2) and Cys^131^ in T7 lysozyme [40] were substituted from His^18^ to Val^191^ and from Cys^131^ to Ser^303^ in *H. parallela* PGRP-SC1 (Fig. 2b). The results of Pro-CHECK showed that 3D model of PGRP-SC1, PPAE-I and GST were all reasonably constructed (Supplementary Fig. S1). Among them, the 3D structure of PGRP-SC1 indicated that three peripheral *α*-helices and five *β*-strands constitute the active domain center (Fig. 2c), and binding pocket was found in the protein surface (Fig. 2d). The phylogenetic analysis showed that *H. parallela* PGRP-SC1 (Hp SC1) clustered with *O. taurus* PGRP 2 (Ot 2) (Fig. 2e).

**Fig. 3:**
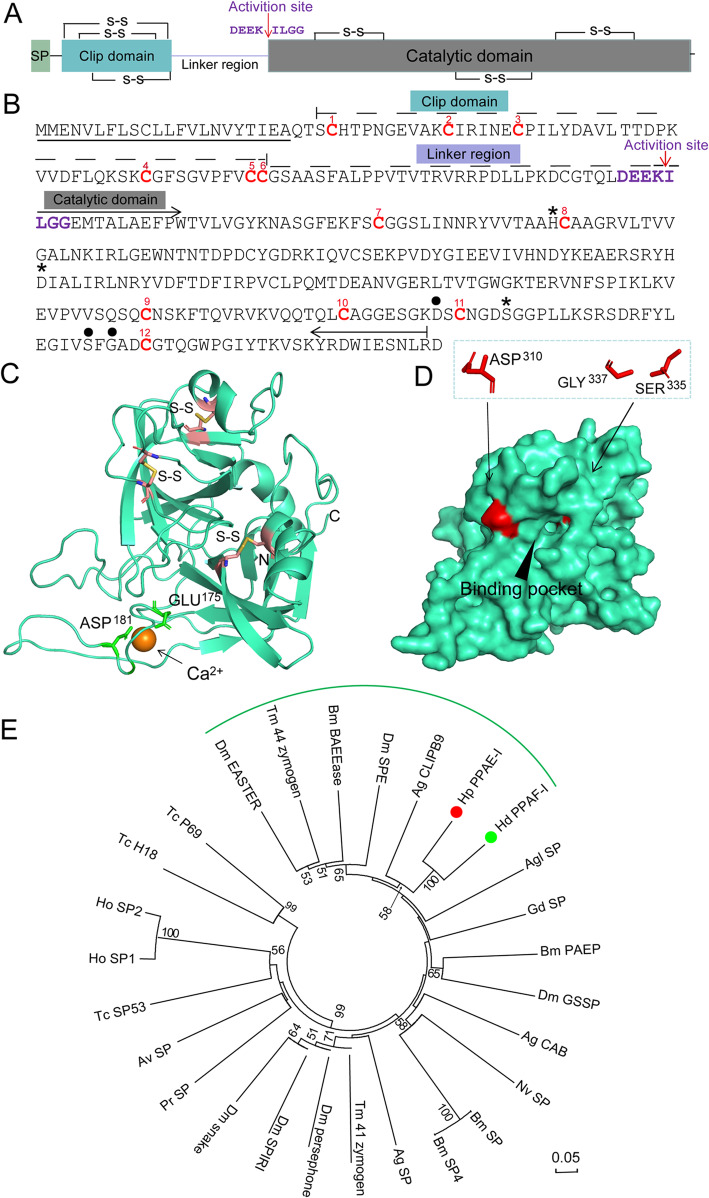
Bioinformatics analysis of *Holotrichia parallela* PPAE-I. **a** Schematic representation of *H. parallela* PPAE-I. Signal peptide (SP) are indicated in green boxes. The disulfide linkages are shown in lines with a symbol (S-S). The position of the peptide bond cleaved during activation is indicated by red arrow. **b** Sequence analysis of *H. parallela* PPAE-I. The predicted secretion signal peptide is underlined. The domain divisions (one clip domain and one catalytic domain) are indicated above the sequence. The absolutely conserved Cys residues in clip domain and catalytic domain are numbered and shown in red. Clip domain is predicted to form three disulfide bonds (1–5, 2–4, 3–6). Catalytic domain is also predicted to form three disulfide bonds (7–8, 9–10, 11–12). The residues of the catalytic triad (His^155^, Asp^221^, Ser^316^) are indicated by asterisks. The important determinants of the specificity pocket in the catalytic domain (Asp^310^, Ser^335^, G1y^357^) are marked by black circles. The potential cleavage activation sites are shown in purple and labeled by red arrow. **c** The overall structure of *H. parallela* PPAE-I. The N-terminus (N), C-terminus (C), calcium ion and disulfide bridges are labeled. **d** Surface of *H. parallela* PPAE-I structure. Substrate binding sites are colored red. **e** Phylogenetic analysis of *H. parallela* PPAE-I. The red and green circles highlight *H. parallela* PPAE-I and the closely homologous specie SP, respectively. Scale bar, 0.05 substitutions per site

Cleavage activation of pro-phenoloxidase mediated by clip domain SPs is critical to melanization, which could originate from melanin to promote wound healing, epidermal sclerosis, free radicals, and the aggregation and encapsulation of pathogens [41]. Sequence analysis showed that the open reading frame of *H. parallela* PPAE-I (a typical SP) contains 1095 nucleotides and 365 amino acids. We found that PPAE-I possessed a catalytic triangle composed of His^155^, Asp^221^ and Ser^316^. PROSITE analysis showed that PPAE-I was composed of a signal peptide (22 amino acid residues), a clip domain (51 amino acid residues) and a catalytic domain (255 amino acid residues). PPAE-I contains 12 extremely conserved cysteine residues and 6 in each of the two domains, which can form intramolecular disulphide bonds (Fig. 3a). In addition, the cutting site of PPAE-I was DEEK^ILGG, where it was recognized and activated by the upstream protease (Fig. 3b). The three highly conserved amino acids Asp^310^, Ser^335^ and G1y^337^ made up the active pocket of the PPAE-I substrate, which determines the specificity of PPAE-I substrate recognition (Fig. 3d). A calcium ion was anchored by Glu^175^ and Asp^183^, which may stabilize the overall structure of the PPAE-I domain (Fig. 3c). Further phylogenetic tree analysis showed that *H. parallela* PPAE-I (Hp PPAE-I) clustered close to *Holotrichia diomphalia* PPAF-I (Hd PPAF-I) with bootstrap values of 100 (Fig. 3e).

**Fig. 4:**
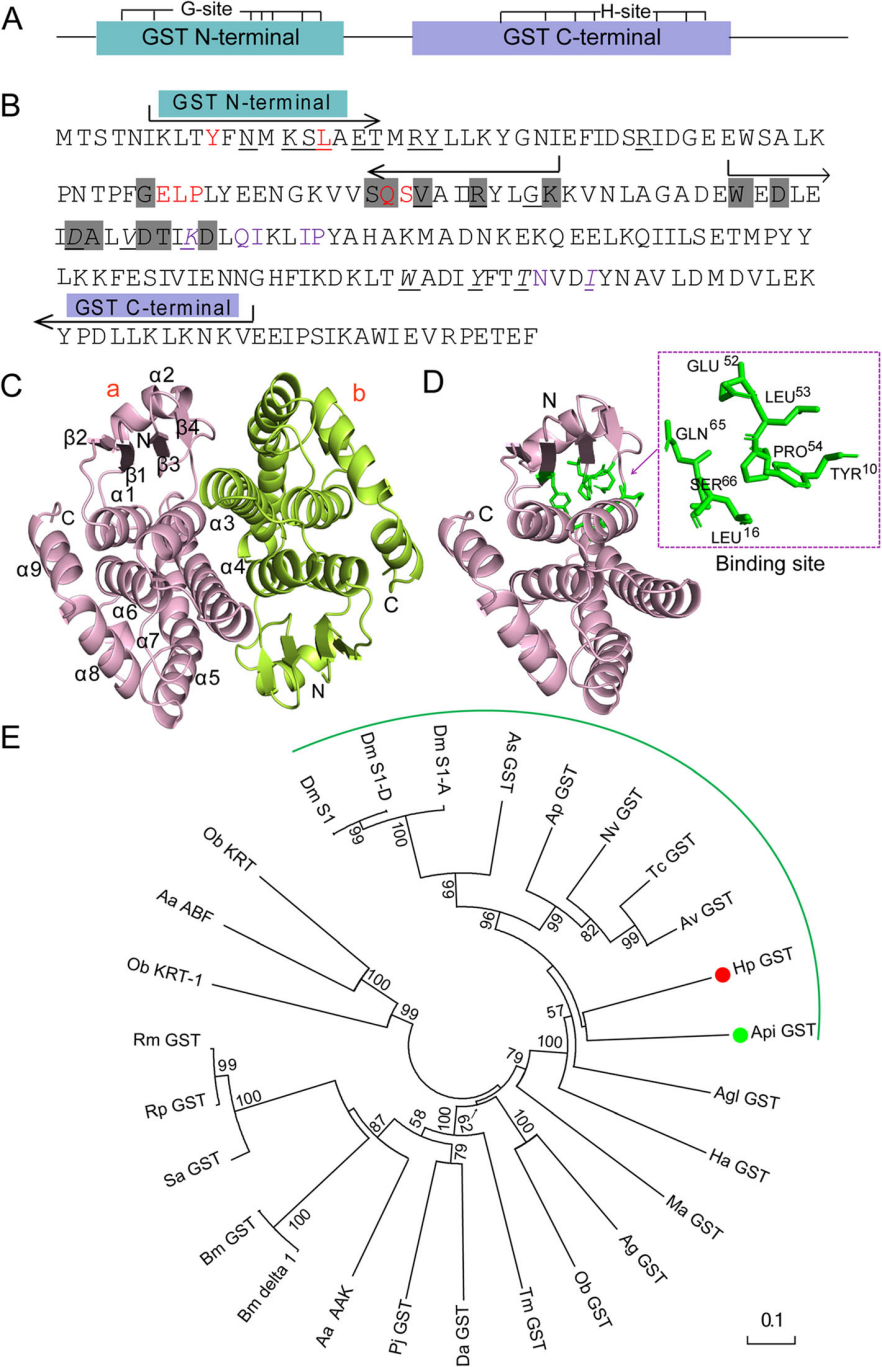
Bioinformatics analysis of *Holotrichia parallela* GST. **a** Schematic representation of *H. parallela* GST. The GST N-terminal and GST C-terminal are indicated in wathet blue and purple boxes, respectively. The G-site and H-site are shown above the boxes. **b** Sequence analysis of *H. parallela* GST. The GST N-terminal and GST C-terminal are indicated above the sequences. The GSH binding sites (G-site) are shown in red. The dimer interface (polypeptide binding site) is shown in dark grey. The C-terminal domain interface is underlined. The substrate binding pocket (H-site) is shown in purple. The N-terminal domain interface (polypeptide binding site) is underlined and tilted **c** The overall structure of *H. parallela* GST. The N-terminus (N), C-terminus (C), *α*-helices and *β*-strands are labeled. ‘a’ and ‘b’ means the GST monomer, respectively. **d** The overall structure of *H. parallela* GST monomer. GSH binding sites are colored green. **e** Phylogenetic analysis of *H. parallela* GST. The red and green circles highlight *H. parallela* GST and the closely homologous specie GST, respectively. Scale bar, 0.1 substitutions per site.

GSTs are the superfamily of multifunctional detoxification isoenzymes involved in the regulation of redox homeostasis and play crucial roles in innate immunity [42]. Sequence analysis showed that *H. parallela* GST genes possessed N/C-terminal domains, G/H-binding sites and dimer interfaces (polypeptide binding sites) (Fig. 4a-b). The GST structure exhibited the overall folding of sigma-class*.* Each monomer of *H. parallela* GST included nine *α*-helices and four *β*-strands (Fig. 4c). One molecule of glutathione (GSH) bound to each *H. parallela* GST monomer and the binding site was located in the deep cleft between the two domains (Fig. 4d). Phylogenetic tree analysis indicated that most species were well differentiated with high bootstrap values, and *H. parallela* GST (Hp GST) clustered close to *A. pisum* GST (Api GST) (Fig. 4e).

### Spatiotemporal analysis of PGRP-SC1, PPAE-I and GST

**Fig. 5:**
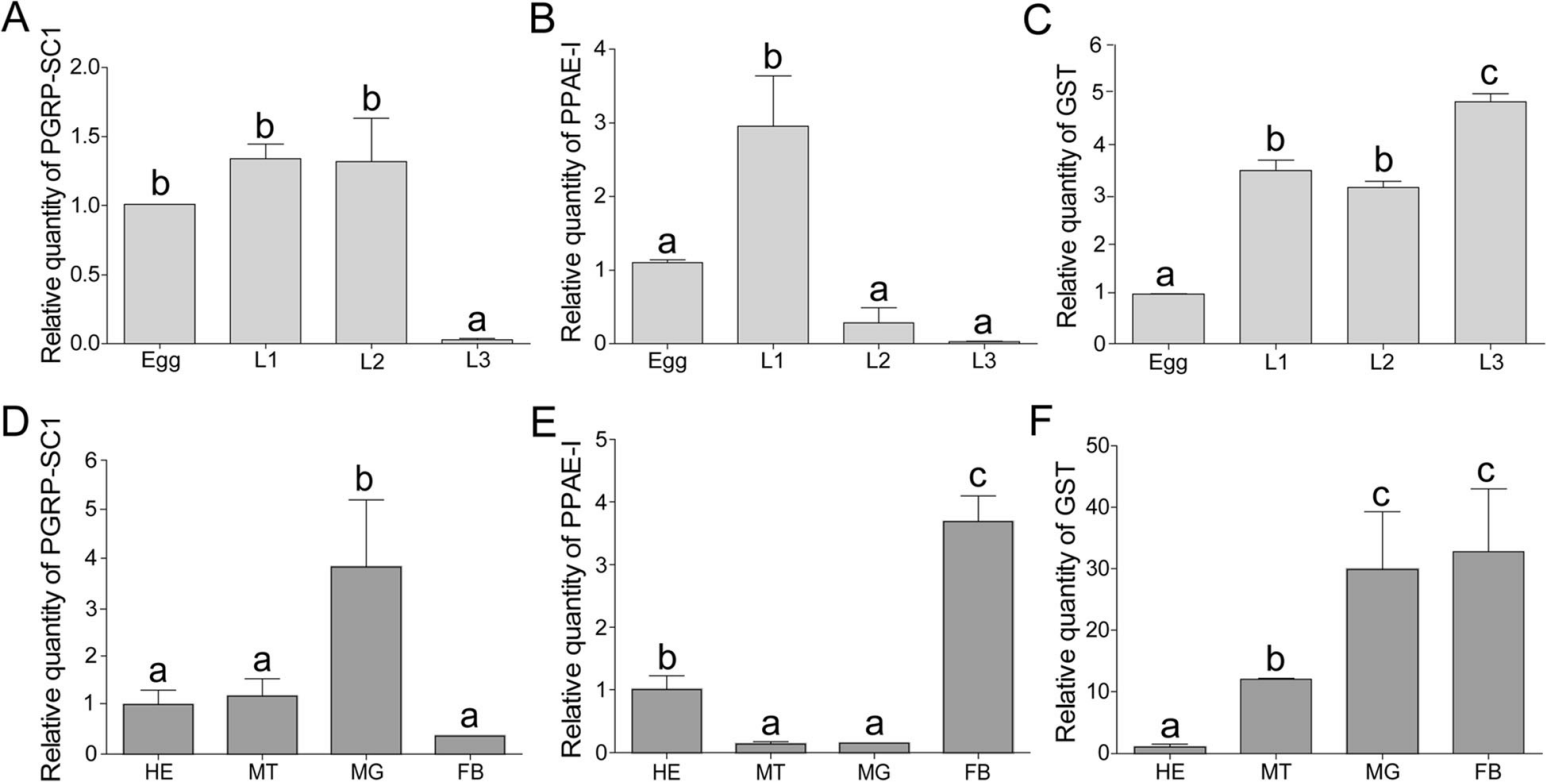
Spatiotemporal expression of *Holotrichia parallela* PGRP-SC1, PPAE-I and GST. The relative expression level of target genes was calculated with the 2^-ΔΔCt^ method. *H. parallela* DAPDH was used as an internal standard to normalize the expression level. **a-c** The developmental expression patterns of *H. parallela* PGRP-SC1, PPAE-I and GST. The relative expression level of target transcripts in the egg served as the calibrator for the developmental expression profiling. Egg (5 days old), L1 (first instar larvae, 10 days old), L2 (second instar larvae, 18 days old), L3 (third instar larvae, 28 days old). **d-f** The tissue-specific expression patterns of *H. parallela* (10-day-old larvae) PGRP-SC1, PPAE-I and GST in hemolymph (HE), malpighian tubule (MT), midgut (MG) and fat body (FB), respectively. The relative expression level of target transcripts in hemolymph were employed as the calibrator for the tissue-specific expression profiling. Different letters above the bars indicate significant differences at *P* < 0.05 (Tukey’ test)

The expression patterns of PGRP-SC1, PPAE-I and GST genes were further determined in different developmental stages and tissues, respectively. The PGRP-SC1 was expressed throughout all developmental stages, exhibiting a low level at third instar larvae (Fig. 5a). PPAE-I exhibited a high level at first instar larvae (Fig. 5b). GST exhibited a high level at third instar larvae (Fig. 5c). For different tissues, PGRP-SC1 and PPAE-I had higher expressions in midgut and fat body, respectively, while GST exhibited high expression in both of them (Fig. 5d-f).

### qRT-PCR validation

**Fig. 6:**
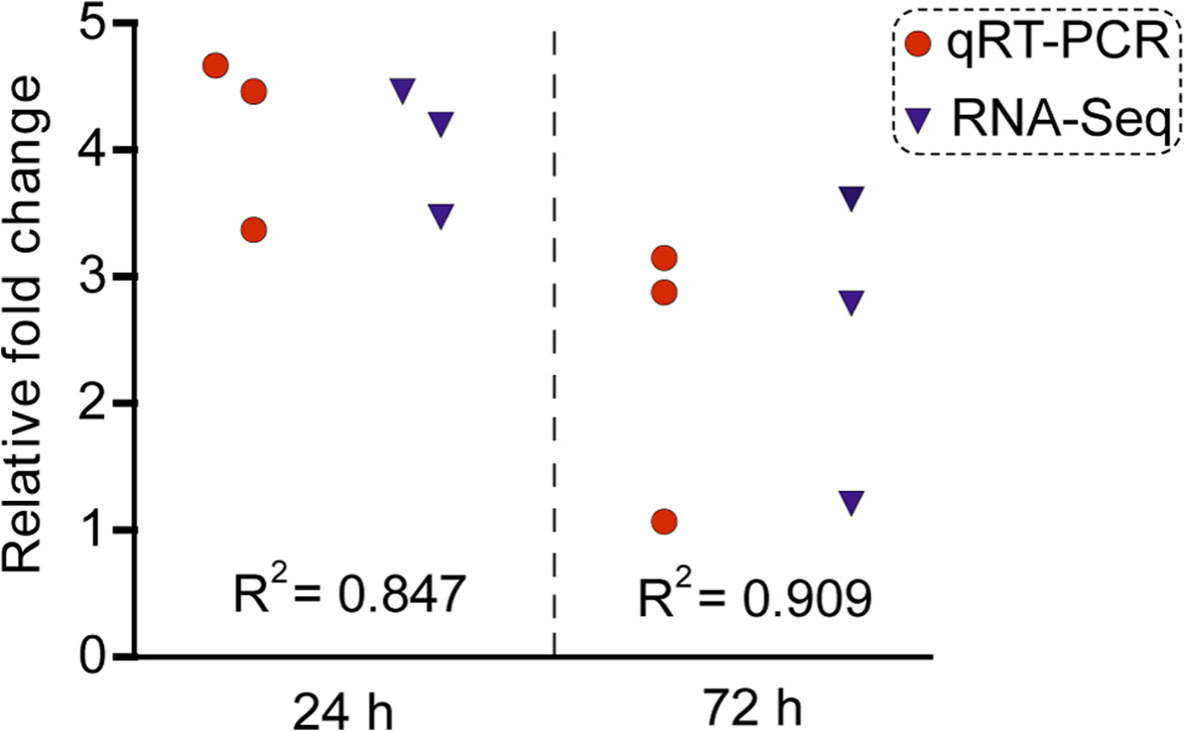
qRT-PCR validation of the transcriptome data in *Holotrichia parallela* larvae postexposure to *Heterorhabditis beicherriana* LF. The relative fold changes in qRT-PCR are presented as the means of three biological replicates. The relative expression level of target genes was calculated with the 2^-ΔΔCt^ method based on the value of water (control) that was ascribed an arbitrary value of 1. *H. parallela* DAPDH was used as an internal standard to normalize the expression level. *R*^*2*^ means the square of correlation coefficient

To validate the transcriptome results, qRT-PCR was performed. The qRT-PCR data shown the PGRP-SC1, PPAE-I and GST gene had a higher expression in the larvae of *H. parallela* treated with nematodes for 24 h with 4.66-, 3.37- and 4.40-fold, respectively, compared to the larvae treated with sterile water. After the larvae treated with nematodes for 72 h, the fold changes were 2.92-, 1.06- and 3.00-fold, respectively (Fig. 6). The results obtained through qRT-PCR were in agreement with the transcriptome data on the fold changes of three candidate immune genes (*R*^*2*^ = 0.847, *P* = 0.194 for 24 h and *R*^*2*^ = 0.909, *P* = 0.256 for 72 h at 95% confidence interval). The consistency of qRT-PCR results with transcriptome data confirmed the reliability and accuracy of sequencing results.

### The expression of PGRP-SC1, PPAE-I and GST after RNAi

**Fig. 7:**
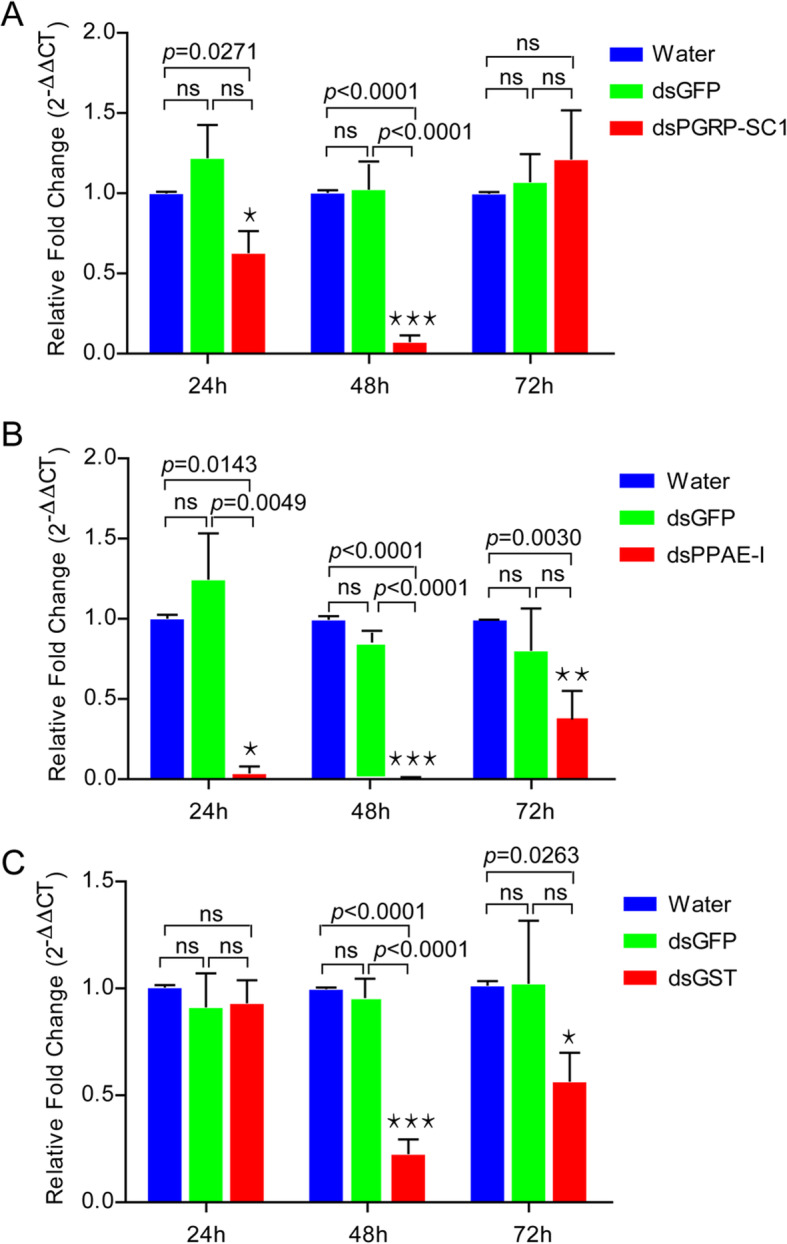
Relative expression level of PGRP-SC1, PPAE-I and GST after RNAi. Data are means ± SEM of three biological replicates, and the relative expression was calculated using the 2^-ΔΔCt^ method based on the value of water (control) at 24, 48, and 72 h that was ascribed an arbitrary value of 1. Statistical differences were determined by multiple *t*-test at the significance levels set at *P < 0.05, **P < 0.01, ***P < 0.001, NS, not significant

RNAi was used to study the functions and their effects on nematode infection resistance of PGRP-SC1, PPAE-I and GST. qRT-PCR confirmed that we successfully knocked down the genes following injection of dsRNAs into the second instar larvae of *H. parallela* (Fig. 7). The expression difference were not significant between control groups (dsGFP injected and water-injected) of those genes in different treatments. However, the expression of PGRP-SC1 and GST in the treatments (dsPGRP-SC1 and dsGST injected larvae) was both significantly reduced in comparison to the water injected larvae at 48 h. The relative expression levels of these two genes were depressed by 92.18% (PGRP-SC1) and 77.00% (GST), respectively. The expression of PPAE-I in dsPPAE-I injected larvae was significantly depressed in comparison to the water injected larvae at 24, 48 and 72 h. The relative expression levels of PPAE-I were depressed by 95.83, 99.10 and 66.33%, respectively.

### Susceptibility to *Heterorhabditis beicherriana* LF after RNAi

**Fig. 8:**
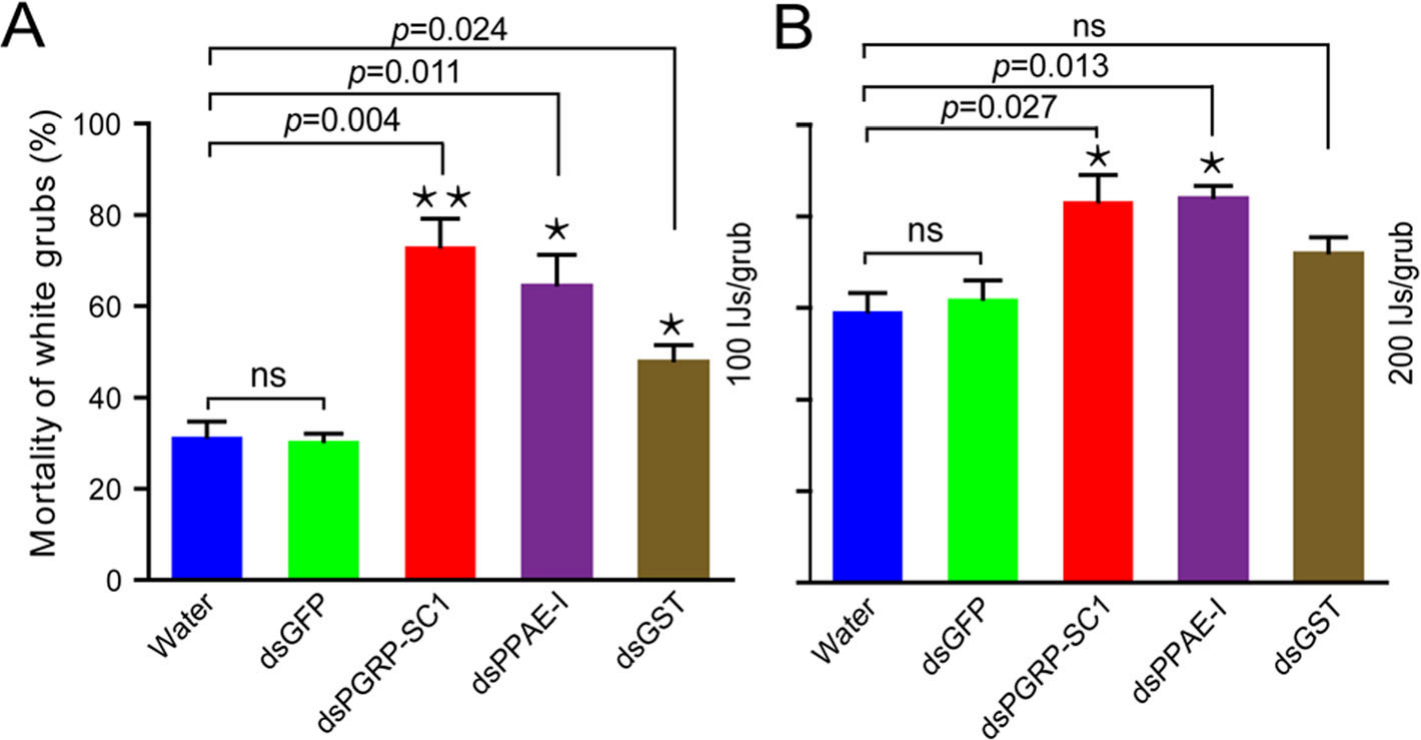
The mortality of white grubs exposed to *H. beicherriana* LF for 24 h (A: 100 IJs/grub; B: 200 IJs/grub) after dsRNA injection. Data are means ± SEM. Statistical differences were determined by multiple *t*-test at the significance levels set at *P < 0.05, **P < 0.01, ***P < 0.001, NS, not significant

The susceptibility of *H. parallela* second instar larvae was investigated after we silenced PGRP-SC1, PPAE-I and GST (Fig. 8). After injection of dsPGRP-SC1, dsPPAE-I or dsGST for 48 h, the larvae were exposed to nematodes (100 and 200 IJs/grub)-incorporated diets for 24 h, and the larvae exhibited a higher mortality than the control larvae injected with dsGFP and water. While dsGFP did not show significant differences to water control, the mortality of *H. parallela* larvae injected with dsPGRP-SC1 or dsPPAE-I was significantly higher than water injected control by increased 41.66% or 33.33% when exposed to nematodes at 100 IJs/grub and 24.08% or 25.00% at 200 IJs/grub. The mortality of *H. parallela* larvae was significantly different between the injection of dsGST and water injected control by increased 16.67% when exposed to nematodes at 100 IJs/grub, whereas the larvae only exhibited a slightly higher mortality by increased 12.97% at 200 IJs/grub.

## Discussion

As one of the most important destructive belowground herbivores in China, the larvae of *H. parallela* have caused considerable damage to crop production [1]. To date, many studies have proven that EPNs are compatible alternatives to conventional insecticides for the larvae of *H. parallela* because they not only can actively search for their hosts in soil but also possess the property of fast-acting than other biological agents [12, 43]. Meanwhile, combating to the host immune response are regarded as crucial to successful parasitism [28]. Thus, the immune and anti-immune mechanisms between *H. parallela* and EPNs are urgently to clarify. In this study, we carried out RNA-Seq to screen the DEGs involved in the immune response of *H. parallela* against EPNs infection and functionally validated three crucial immune-related genes using in vivo RNAi technology.

Through comparative analysis, 925 transcripts were induced upon nematode challenge for 24 h in our grubs, whereas only 314 genes were induced in *H. virescens* larvae after exposure to *H. bacteriophora* for 23 h [39]. However, homologue of thioester-containing complement protein 3, basement membrane component (glutactin) and recognition protein (GNBP-like 3) which were not identified in the 74 DEGs implicating in immune responses of *H. parallela* has been proven to be involved in the immune responses of *Drosophila* larvae against *H. bacteriophora* (H222 strain) infection [38]. Therefore, we think the immune responses between white grubs and *Drosophila* against nematodes infection were different. In addition, some sequences respond to symbiotic bacterium were detected after treatment with nematodes for 72 h (Supplementary Table S2). Previous studies have shown that 10% of *H. bacteriophora* can escape the encapsulation of *P. japonica* larvae after entering for 24 h [44] and the escaped nematodes will release symbiotic bacteria 30 min later [45]. Thus, the symbiotic bacteria of *H. beicherriana* LF was predicted to participate in immune responses after *H. parallela* larvae treated with the nematode for 72 h.

Identification of parasite/pathogen invasion in the host is the initial step in the initiation of immune responses, and this process relies on biosensor proteins called pattern recognition receptors (PRRs), which can detect and bind to certain pathogen-associated molecular patterns (PAMPs) on the surface of invading microbes or foreign bodies [46, 47]. PGRPs are important members of insect PRRs and play an important role in innate immunity [48, 49]. Previous studies have shown that PGRP-LB1, −LB2, −LC2, −LC3 and -LD in *Monochamus alternatus* are likely to encode membrane proteins [50]; PGRP-SC1 of *H. parallela* contains a similar structure (without a signal peptide but containing a transmembrane segment), indicating that it may function on the cell membrane to recognize and trigger immune responses. Moreover, 2 of 5 key residues responsible for zinc binding and amidase catalytic activity were substituted, indicating that *H. parallela* PGRP-SC1 has no amidase activity and cannot cleave the bacterial peptidoglycan (PGN) [51]. However, this type of PGRP may function as a sensor for ligand-dependent signalling to activate the pro-phenoloxidase (PPO) cascade [52]. For example, overexpression of PGRP-LE in *D. melanogaster* led to the activation of the PPO cascade [53]. Therefore, we speculate that *H. parallela* PGRP-SC1 may participate in the PPO cascade reaction as a signal recognition receptor. Further phylogenetic analysis of PGRP showed that *H. parallela* PGRP-SC1 and *B. mori* PGRP-L1 were in a cluster. It is already known that PGRP-L1 in *B. mori* could induce the expression of antigen processing machinery (APM) genes and may be players in the immune deficiency (IMD) pathway [54]. Therefore, we speculate that *H. parallela* PGRP-SC1 may also be involved in the IMD pathway. However, the detailed functionality of PGRP-SC1 in *H. parallela* merits further studies with more biochemical methods.

Once the insect PRRs recognize the pathogen, the second step of innate immune-signal modulation is immediately initiated. At this time, the cascade reaction of SPs mainly relies on continuously expanding signals and eventually transmitting the signals to the nucleus [55]. Our research indicated that *H. parallela* PPAE-I was a typical serine protease with a clip domain and a catalytic domain, and further phylogenetic tree analysis showed that *H. parallela* PPAE-I clustered close to *H. diomphalia* PPAF-I. Previous findings have demonstrated that clip domain SPs are actively involved in the prophenoloxidase-activation cascade [56, 57]. Moreover, *H. diomphalia* PPAF-I could activate *H. diomphalia* PPO in the presence of PPAF-II [58]. In summary, *H. parallela* PPAE-I may function in inducing pro-phenoloxidase activity and mediating the melanization response.

GST is a widespread multigene family of detoxification enzymes and acts as an effector molecule after induction by immunization in innate immunity [59]. GST has a variety of functions, including detoxification of endogenous and/or xenobiotic compounds and protection of cells from oxidative damage, whereas other functions may include contributing to metabolic and signalling pathways [60, 61]. A number of studies have shown that GST is generally induced by immunization and acts as an effector to regulate the metabolism of exogenous toxic substances. For instance, *T. castaneum* GST was activated to defend against lipopolysaccharide (LPS)-challenge [62], and *P. brevitarsis* GST was upregulated in response to *E. coli* challenge [24]. In addition, sequence analysis showed that *H. parallela* GST possessed the characteristics of the GST family, and phylogenetic analysis indicated that *H. parallela* GST and *D. melanogaster/N. vespilloides* GST were in a cluster, which has been proven to respond to microbial challenge [63, 64]. In general terms, *H. parallela* GST may play a partial immune protection role in *H. parallela* against nematode infection.

Then, the roles of these three genes were determined by dsRNA injection-mediated RNAi and pathogenicity assays. The results showed that silencing of PGRP-SC1 and PPAE-I resulted in significant susceptibility of *H. parallela* to nematodes, and the mortality of *H. parallela* larvae was increased to different degrees compared to that of the control (water-injected). In addition, silencing of GST also resulted in increased susceptibility of *H. parallela* to nematodes. These results suggest that PGRP-SC1, PPAE-I, and GST are involved in immune responses to resist nematode infection.

## Conclusions

Overall, our study strongly support that PGRP-SC1, PPAE-I and GST are genes related to nematode resistance in *H. parallela*, and the detailed functionalities of these genes deserve further investigation. Meanwhile, these results may help to elucidate the nematode resistance mechanism and provide valuable insights in designing appropriate resistance management strategies for this famous agricultural pest. In addition, more studies are needed to elucidate the immune mechanisms between *H. parallela* and symbiotic bacteria of *H. beicherriana* LF or other kinds of EPNs.

## Methods

### Nematode and insect culture

Entomopathogenic nematode *H. beicherriana* LF were cultured in vivo on larvae of the greater wax moth (*Galleria mellonella*) at 25 °C and 80% relative humidity (RH). Released infective juveniles were collected and stored at 7 ± 1 °C for no more than 15 days before use [65]. The larvae of *H. parallela* were fed germinated wheat seeds, and actively feeding grubs (approximately 13 days old and at the beginning of second instar) were kept individually in the holes of six-well bioassay trays (Corning Inc., NY, USA) filled with 15 g and 18% soil moisture (w/w) of high temperature sterilized sandy loam soil (76% sand, 16% silt, 6% clay, 0.5% organic matter; pH 6.7) for use.

### Nematode infection to *Holotrichia parallela*

One hundred IJs were added into each hole of the six-well bioassay trays described above. A total of 60 *H. parallela* larvae were treated with nematodes, and control group was treated with sterile water. The six-well bioassay trays were then transferred into a dark incubator under the following conditions: 25 °C and 80% RH. The midgut samples (*n* = 3 for each treatment) of *H. parallela* used for RNA-Seq were collected in RNase-free tubes at 24 h and 72 h after treatment with nematodes or sterile water following the method below.

### Disintegration of *Holotrichia parallela* midgut

*H. parallela* larvae treated with nematodes or sterile water were transferred on ice for 1 min and then placed on a precooled petri dish (*d* = 15 cm). After adjusting the focal length of SMZ-168 Stereo Zoom microscope (Sinolab Inc., Beijing, China) until the insect body could be observed clearly, the head and tail of the larvae were cut off with scissors and the whole midgut was carefully pulled out using tweezers. The malpighian tubule and fat around the midgut were removed with tweezers, and the peritrophic membrane was pulled to remove all food particles in the midgut. After three rinses with RNase-free water, three samples for each treatment were collected in a 1.5 ml RNase-free tube and stored at − 80 °C for RNA isolation. The experiment was performed with two replicates of each treatment for RNA-Seq.

### RNA isolation and cDNA library preparation

Total RNA was extracted using TRIzol Reagent (Invitrogen, Carlsbad, CA, USA) following the manufacturer’s protocol followed by ethanol/isopropanol precipitation and suspended in a final volume of 30 μl with RNase-free water. After extraction, the total RNA quality of different treatments were measured with NanoDrop 2000 spectrophotometer (Thermo Scientific) and in a 1.5% agarose gel. RNA integrity and concentration were subsequently analyzed using 2100 RNA Nano 6000 Assay Kit (Agilent Technologies, CA, USA).

Following the Illumina protocols, 2 μg RNA of different treatment was used for cDNA library construction, and each treatment was replicated two times. Following the use of oligo (dT) magnetic beads, fragmentation of mRNA, generation of the first strand cDNA with a random hexamer primer and synthesis of second strands, cDNA end repair and adenylation at the 3′ end, and adapter ligation and cDNA fragment enrichment were performed. Then, the cDNA products were purified and amplified by PCR to create the final cDNA library.

### RNA-Seq and de novo assembly

Paired-end sequencing was conducted on an Illumina HiSeq 2000 (Illumina Inc., USA) at Anoroad Genomics Co., Ltd. (Beijing, China). After sequencing, the raw reads were filtered to generate clean reads. First, the reads with adapters were removed, and then the reads with > 5% unknown bases (N) were discarded. Finally, the low-quality reads (with > 50% of nucleotides for which the Phred Quality Score Q was less than or equal to 19) were filtered out.

The clean reads of each treatment were loaded to Trinity (Trinity Release v2.4.0) [66] for de novo assembly under the paired-end mode with default parameters. Based on the filtered clean data, the full-length transcript sequence was assembled with Trinity, and each gene was taken based on the transcript sequence. The longest transcript sequence was recorded as ‘Unigene’.

RPKMs (Reads Per Kilobase Million Mapped Reads) [67] were used in DESeq2 [68] to compare the differences of gene expression between the nematodes and sterile water treat. Genes with |log2 Fold Change| ≥ 1 and q < 0.05 were selected as significantly DEGs. Then, DEGs were further annotated by Gene Ontology (GO) function (http://www.geneontology.org/).

### Identification of immune-related genes in *Holotrichia parallela*

To identify the immune-related genes involved in the process of *H. parallela* larvae defence against nematode infection, we query with the terms that are reported as the immune relative genes from the differential gene expression data that obtained above. The terms mainly include ‘immune’, ‘recognition protein’, ‘haemocyte’, ‘glutactin’, ‘antimicrobial’, ‘lysozyme’, ‘lipopolysaccharide’, ‘lectins’, ‘peptidoglycans’ etc. All candidate immune genes were manually verified with the BLASTx program at the National Center for Biotechnology Information (NCBI: https://www.ncbi.nlm.nih.gov/). Thereafter, we focused on immune genes that were significantly induced at both time points, as these potential candidate immune-related genes may be involved in the immune responses to effectively resist nematode infection.

### Bioinformatics analysis of three potential candidate immune-related genes

The deduced amino acid sequences of three potential candidate immune-related genes (PGRP-SC1, PPAE-I and GST) were obtained using the Translate tool provided by the Swiss Institute Bioinformatics (https://web.expasy.org/translate/). The signal peptide and transmembrane domain were analyzed by SignalP 5.0 (http://www.cbs.dtu.dk/services/SignalP) and TMHMM server v. 2.0 (http://www.cbs.dtu.dk/services/TMHMM/). In addition, domain analysis of the retrieved protein sequences were executed by NCBI Conserve-Domain Tool (https://www.ncbi.nlm.nih.gov/cdd) and PROSITE (http://au.expasy.org/prosite/). The 3D structures were obtained using templates of *Bumblebee* PGRP-SA (5xz4.1.A, 34.76% identity), *Holotrichia diomphalia* PPAF-I (2olg.1.A, 66.67% identity) and *Nilaparvata lugens* GST S2 (5h5l.1.A, 40.30% identity), respectively, employing online Swiss-model software. Pro-CHECK (http://servicesn.mbi.ucla.edu/PROCHECK/) was used to evaluate the rationality of the 3D structure of the constructed model. The generated PDB files were then visualized with Pymol Molecular Graphics System.

Phylogenetic analysis of PGRP-SC1 from *H. parallela* and 53 PGRPs from 12 other insect species; PPAE-I from *H. parallela* and 26 SPs from 12 other insect species; GST from *H. parallela* and 26 GSTs from 12 other insect species, which were used to construct a phylogenetic tree using MEGA5.0. Phylogenetic analysis were conducted by the neighbour-joining method with *p*-distance under the default parameters. Bootstrap values were obtained by the bootstrap method using 1000 repetitions. The species name, gene name, abbreviation and GenBank accession involved in phylogenetic analysis are listed in Supplementary Table S3.

### Spatiotemporal analysis of PGRP-SC1, PPAE-I and GST

To determine the expression profiles of these genes in different development stages and tissues, we collected eggs (5 days old, ~ 60 mg), first instar larvae (10 days old, ~ 60 mg), second instar larvae (18 days old), third instar larvae (28 days old) and various tissues (hemolymph, malpighian tubule, midgut and fat body) of the first instar larvae for RNA isolation. Total RNA samples of each treatment were individually extracted using TRIzol Reagent (TIANGEN, Biotech (Beijing) Co., Ltd., China) and each treatment repeat three times. First-strand cDNA was synthesized from 1 μg of total RNA following the instructions of TransScript One-Step gDNA Removal and cDNA Synthesis SuperMix (TransGen Biotech (Beijing) Co., Ltd., China). The cDNA products were diluted 10-fold for use as template in qRT-PCR.

qRT-PCR was performed with Tip Green qPCR SuperMix (TransGen, China) on an ABI Prism 7500 Fast Detection System using a GO Taq®qPCR kit (Invitrogen, USA) according to the manufacturer’s instructions. Gene-specific primers designed for target genes are listed in Supplementary Table S4. qRT-PCR reactions were performed in a 20 μl mixture containing 2 μl of cDNA, 10 μl of Tip Green qPCR SuperMix, 0.4 μl of each primer (10 μM), and 7.2 μl of nuclease-free water. The optimized qRT-PCR programme conditions were 94 °C for 30 s followed by 40 cycles of 94 °C for 5 s and 60 °C for 34 s. After the cycling protocol, melting curves were obtained by increasing the temperature from 60 to 95 °C (0.2 °C sec^− 1^) to denature the double-stranded DNA. *H. parallela* DAPDH was used as an internal standard to normalize the expression level. Each qRT-PCR experiment was performed with three biological replicates and four technical replicates. The relative expression level of target genes was calculated with the 2^-ΔΔCt^ method [69].

### qRT-PCR validation

To confirm the transcriptome results, these immune-related genes (i.e., PGRP-SC1, PPAE-I and GST) of *H. parallela* responding to nematode infection were selected for qRT-PCR analysis. Fresh total RNA was obtained as described in RNA-Seq and qRT-PCR was performed as described above.

### RNA interference to *Holotrichia parallela* larvae

dsRNA synthesis: RNAi was used to study the functions of PGRP-SC1, PPAE-I, and GST involved in the immune defence system of *H. parallela* against *H. beicherriana* LF infection. Specific primers (Supplementary Table S4) with T7 promoter sequences were designed to synthesize dsRNA of PGRP-SC1, PPAE-I, GST and GFP using the T7 RiboMAX™ Express RNAi kit (Promega, WI, USA) according to the manufacturer’s instructions. The size of the dsRNA products was confirmed by electrophoresis on 1.2% agarose gel, and the final concentration of dsRNA was adjusted to 1 μg/μl.

In vivo RNAi: For in vivo RNAi, 1.5 μg of each dsRNA, dsGFP and the same volume of RNase-free water were injected into the haemocoel of *H. parallela* second instar larvae (18–20 days old) through the abdomen (WPI microinjection system, USA). After injection, the larvae were kept individually in the holes of six-well bioassay trays and fed germinated wheat seeds. The six-well bioassay trays were transferred into a dark incubator under the following conditions: 25 °C and 80% RH. The midgut samples (*n* = 3 for each treatment) from challenged or control groups (dsGFP-injected and water-injected) was collected after treatment for 24, 48 and 72 h, respectively, and the experiment was performed with three biological replicates. The qRT-PCR process was executed as described above.

### Susceptibility of *Holotrichia parallela* larvae to *Heterorhabditis beicherriana* LF after RNAi

Susceptibility of *H. parallela* larvae to *H. beicherriana* LF after RNAi of PGRP-SC1, PPAE-I and GST was conducted by determining the pathogenicity change of *H. beicherriana* LF on *H. parallela* larvae. After injection following our previous procedures, the larvae (18–20 days old) were kept individually in the holes of six-well bioassay trays, fed germinated wheat seeds and then transferred into a dark incubator as described above. Forty eight hours later, two concentrations (100 and 200 IJs/grub) of *H. beicherriana* LF were added to each hole of six-well bioassay trays, respectively. Each injection treatment consisted of 36 individuals (grub) and the experiment was performed three times. The grub mortality was monitored after 24 h, and the infected grubs were marked as inactive and red.

### Statistical analysis

Data statistics were performed using GraphPad Prism software version 8.0. All proportions were transformed by arcsine square root transformation in Microsoft Excel 5.0 before analysis [70]. The significance levels of spatiotemporal analysis were analyzed using one-way analysis of variance (ANOVA) followed by Tukey significant difference test (*P* < 0.05). The significance levels of in vivo RNAi and white grubs mortality were analyzed using multiple *t*-test at the significance levels set at *P < 0.05, **P < 0.01, ***P < 0.001, NS, not significant.

## Supplementary Information

**Additional file 1: Table S1.** The annotations of PGRP-SC1, PPAE-I and GST. **Table S2:**. The annotations of symbiotic bacteria in the transcriptomes of the *Holotrichia parallela* larvae postexposure to *H. beicherriana* LF for 72 h. **Table S3.** Summary of all species name, gene name, abbreviation and GenBank accession involved in phylogenetic analysis. **Table S4.** Oligonucleotides used for qRT-PCR and RNAi. The T7 promoter sequence is bolded in RNAi. **Fig. S1.** Rationality evaluation results of PGRP-SC1 (a), PPAE-I (b) and GST (c) 3D model. The red regions (A, B, L) represents the residues in most favoured regions; Bright yellow regions (a, b, l, p) represents the residues in additional allowed regions; Dark yellow regions (~a, ~b, ~l, ~p) represents the residues in generously allowed regions.

## Data Availability

These sequence data of *H. parallela* PGRP-SC1, PPAE-I and GST have been submitted to the GenBank databases under accession number DN15104, DN15190 and DN16733, respectively.

## Abbreviations

AMPs: Antimicrobial peptides
ANOVA: Analysis of variance
APM: Antigen processing machinery
ATP: Adenosine triphosphate
CCOB: Cellular component organization or biogenesis
DEGs: Differentially expressed genes
EPNs: Entomopathogenic nematodes
FB: Fat body
GO: Gene ontology
GST: Glutathione s-transferase
HE: Hemolymph
HSPs: Heat shock protein
IJs: Infective juveniles
IMD: Immune deficiency pathway
LPS: Lipopolysaccharide
MG: Midgut
MT: Malpighian tubule
NCBI: National Center for Biotechnology Information
PAMPs: Pathogen-associated molecular patterns
PGN: Peptidoglycan
PGRP: Peptidoglycan recognition protein
PGRP-SC1: Peptidoglycan recognition protein SC1
PPAE-I: Pro-phenoloxidase activating enzyme-I
PPICST: Presynaptic process involved in chemical synaptic transmission
PPO: Prophenoloxidase
PRPs: Pattern recognition proteins
PRRs: Pattern recognition receptors
qRT-PCR: Quantitative real-time PCR
RH: Relative humidity
RPKMs: Reads Per Kilobase Million Mapped Reads
SOD: Superoxide dismutase
SP: Signal peptide
SPs: Serine proteases
TM: Transmembrane domain
