# Supplementary material for: Immune-related genes of the larval Holotrichia parallela in response to entomopathogenic nematodes Heterorhabditis beicherriana LF
Source: BMC Genomics. 2021 Mar 17;22:192. doi: 10.1186/s12864-021-07506-4 (PMC7967997; doi:10.1186/s12864-021-07506-4)
Supplement: Supplementary file 1 — Additional file 1: Table S1. The annotations of PGRP-SC1, PPAE-I and GST. Table S2:. The annotations of symbiotic bacteria in the transcriptomes of the Holotrichia parallela larvae postexposure to H. beicherriana LF for 72 h. Table S3. Summary of all species name, gene name, abbreviation and GenBank accession involved in phylogenetic analysis. Table S4. Oligonucleotides used for qRT-PCR and RNAi. The T7 promoter sequence is bolded in RNAi. Fig. S1. Rationality evaluation results of PGRP-SC1 (a), PPAE-I (b) and GST (c) 3D model. The red regions (A, B, L) represents the residues in most favoured regions; Bright yellow regions (a, b, l, p) represents the residues in additional allowed regions; Dark yellow regions (~a, ~b, ~l, ~p) represents the residues in generously allowed regions. [file 12864_2021_7506_MOESM1_ESM.docx]

**Supplementary Tables and Figures**

**Supplementary Table 1.** The annotations of PGRP-SC1, PPAE-I and GST

| **Gene name** | **Length (nt)** | **ORF**  **(aa)** | **Unigene ID** | **Status** | **E-value** | **Matched species** |
| --- | --- | --- | --- | --- | --- | --- |
| PGRP-SC1 | 1755 | 329 | TRINITY_DN15104_c0_g1 | Complete ORF | <0.0 | *Tribolium castaneum* |
| PPAE-I | 1533 | 365 | TRINITY_DN15190_c0_g1 | Complete ORF | <0.0 | *Holotrichia diomphalia* |
| GST | 1008 | 209 | TRINITY_DN16733_c0_g1 | Complete ORF | <0.0 | *Oryctes borbonicus* |

**Supplemental Table 2.** The annotations of transcript sequence respond to symbiotic bacterium after treatment with nematodes for 72 h

| **Unigene ID** | **Description** | **Matched species** |
| --- | --- | --- |
| TRINITY_DN13460_c0_g1 | Angiotensin-converting enzyme-like | *Tribolium castaneum* |
| TRINITY_DN18147_c1_g1 | Ras-related protein Rab-35 | *Tribolium castaneum* |

**Supplemental Table 3.** Summary of all species name, gene name, abbreviation and GenBank accession involved in phylogenetic analysis

| **Protein name** | **Species name and abbreviation** | **GenBank accession** |
| --- | --- | --- |
| PGRP-SC1 | Bm: [*Bombyx mori*] | Bm S1: NM_001043371; Bm S2: AF441723;  Bm S3: JN120254; Bm S5: NP_001036858.1;  Bm S6: XP_012548099.1; Bm L1:XP_004929813.1;  Bm L2: XP_004929814.1; Bm L3: XP_028032135.1;  Bm L4: XP_012549076.1; Bm L5: XP_004929948.1;  Bm L6: XP_004929966.1 |
|  | Ms: [*Manduca sexta*] | Ms1: AF413068; Ms2: ACX49764 |
|  | Dm: [*Drosophila melanogaster*] | Dm LB: NP_001247052.1; Dm LC: NP_729468;  Dm LE: NP_573078; Dm LF: NM_140042;  DmP SA: NP_572727; Dm SB1: NM_140660.4;  Dm SB2: NM_140659.3; Dm SC1: NM_136563.1;  Dm SC2: NP_610410; Dm SD: NM_139888.3 |
|  | Tc: [*Tribolium castaneum*] | Tc SC2: EFA01279.2; Tc LE: EFA01280.1;  Tc SA: XP_969883 |
|  | Ha: [*Helicoverpa armigera*] | Ha B: AFP23116; Ha C: AFP23117 |
|  | Ag: [*Anopheles gambiae*] | Ag 1: XP_314103.4; Ag 2: XP_558600.3;  Ag 3: XP_558599.3; Ag 4: XP_001688527.2;  Ag 5: XP_001688528.2; Ag 6: XP_001688678.1;  Ag 8: XP_003435776; Ag 9: XP_310547.4;  Ag 10: XP_001688526.1 |
|  | Ot: [*Onthophagus taurus*] | Ot 2: XP_022903161.1; Ot LFX4: XP_022914149.1;Ot LFX3: XP_022914140.1; Ot 1: XP_022916766.1;Ot LE: XP_022914682.1 |
|  | Nv: [*Nicrophorus vespilloides*] | Nv LA: XP_017776594.1; Nv LC: XP_017776604.1;  Nv SC2: XP_017776602.1; Nv 3: XP_017785944.1 |
|  | Ld: [*Leptinotarsa decemlineata*] | Ld LC: XP_023026842.1; Ld LA: XP_023018543.1;  Ld LE: XP_023013200.1 |
|  | Ap: [*Agrilus planipennis*] | Ap LE: XP_018318743.1 |
|  | Sz: [*Sitophilus zeamais*] | Sz 1: ABZ80672.1; Sz LBt: QBC16602.1 |
|  | Tm: [*Tenebrio molitor*] | Tm LE: CCV65021.1 |
| PPAE-I | Ag: [*Anopheles gambiae*] | Ag SP: CAB90819.1; Ag CLIPB9: HM070255.1;  Ag CAB: CAB91840.1 |
|  | Bm: [*Bombyx mori*] | Bm BAEEase: ABB58762.1; Bm SP: BAB91156.1;  Bm SP4: NP_001036891.1; Bm PAEP:NP_001036832.1 |
|  | Dm: [*Drosophila melanogaster*] | Dm EASTER: NP_524362.2;  Dm persephone: NP_573297.1;  Dm SPE: NP_651168.1; Dm SPIRI: NP_727276.1;  Dm GSSP: NP_733197.1; Dm snake: NP_524338.2 |
|  | Hd: [*Holotrichia diomphalia*] | Hd PPAF-I: BAA34642.1 |
|  | Tm: [*Tenebrio molitor*] | Tm 41 zymogen: AB363979.1;  Tm 44 zymogen: AB363980.2 |
|  | Nv: [*Nasonia vitripennis*] | Nv SP: NP_001166063.1 |
|  | Gd: [*Galeruca daurica*] | Gd SP: AYN79576.1 |
|  | Pr: [*Pyrocoelia rufa*] | Pr SP: AAN17336.1 |
|  | Av: [*Asbolus verrucosus*] | Av SP: RZC38039.1 |
|  | Agl: [*Anoplophora glabripennis*] | Agl SP: JAB68214.1 |
|  | Tc: [*Tribolium castaneum*] | Tc H18: EEZ99238.2; Tc P69: EFA09211.1;  Tc SP53: KYB25980.1 |
|  | Ho: [*Holotrichia oblita*] | Ho SP1: ACU00114.1; Ho SP2; ACU00115.1 |
| GST | Bm: [*Bombyx mori*] | Bm delta 1: NP_001037183.1; Bm GST: CAA07071.1 |
|  | Dm: [*Drosophila melanogaster*] | Dm S1: NP_001261040.1; Dm S1-A: AAF57901.1;  Dm S1-D: AGB93572.1 |
|  | Aa: [*Aedes aegypti*] | Aa AAK: AAK64286.1; Aa ABF: ABF18476.1 |
|  | Ob: [*Oryctes borbonicus*] | Ob GST: KRT84505.1; Ob KRT: KRT79260.1;  Ob KRT-1: KRT86754.1 |
|  | Tc: [*Tribolium castaneum*] | Tc GST: XP_008190960.1 |
|  | Sa: [*Sitobion avenae*] | Sa GST: AMZ00813.1 |
|  | Rp: [*Rhopalosiphum padi*] | Rp GST: AJN57846.1 |
|  | Ap: [*Agrilus planipennis*] | Ap GST: XP_018334284.1 |
|  | Api: [*Acyrthosiphon pisum*] | Api GST: NP_001313601.1 |
|  | Rm: [*Rhopalosiphum maidis*] | Rm GST: AUG84068.1 |
|  | Tm: [*Tenebrio molitor*] | Tm GST: AIL23556.1 |
|  | As: [*Anopheles sinensis*] | As GST: KFB41358.1 |
|  | Ha: [*Helicoverpa armigera*] | Ha GST: ADI32892.1 |
|  | Da: [*Dendroctonus armandi*] | Da GST: AII79438.1 |
|  | Pj: [*Propylea japonica*] | Pj GST: QFN66798.1 |
|  | Av: [*Asbolus verrucosus*] | Av GST: RZC43019.1 |
|  | Nv: [*Nicrophorus vespilloides*] | Nv GST: XP_017775172.1 |
|  | Ma: [*Mesocricetus auratus*] | Ma GST: NP_001268559.1 |
|  | Ag: [*Anopheles gambiae*] | Ag GST: AAM61893.1 |
|  | Agl: [*Anoplophora glabripennis*] | Agl GST: XP_018573274.1 |

**Supplemental Table 4.** Oligonucleotides used for qRT-PCR and RNAi

|  | **Forward Primer (5’-3’)** | **Revers Primer (5’-3’)** |
| --- | --- | --- |
| **qRT-PCR** |  | |
| PGRP-SC1 | ATCGTCCAGCAACAAGC | GTCGCCGTCAGTATCGT |
| PPAE-I | GAGTGATCCACCGCAAGAGAAC | AAACGGCGAAGTAGCAAAATGT |
| GST | TGAAGAGTCTCGCTGAA | GCAACGCTCTGACTAAC |
| DAPDH | AATACCTTTTAGTGGTCCTTCCG | TGCATGCTATCACAGCTACGC |
| **RNAi** |  | |
| PGRP-SC1  sense strand | GGATCC**TAATACGACTCACTATAGG**  TCCTGTCAACATACAACTTCACC | GTAGGAATCAGGCAGCACGCCCAA |
| PGRP-SC1  antisense strand | GTCCTGTCAACATACAACTTCACC | GGATCC**TAATACGACTCACTATAGG**TAGGAATCAGGCAGCACGCCCAA |
| PPAE-I  sense strand | GGATCC**TAATACGACTCACTATAGG**  TCTCTTAGATTACTCTCAATCCA | GACATTGCCTTAATAAGATTGAAC |
| PPAE-I  antisense strand | GTCTCTTAGATTACTCTCAATCCA | GGATCC**TAATACGACTCACTATAGG**  ACATTGCCTTAATAAGATTGAAC |
| GST  sense strand | GGATCC**TAATACGACTCACTATAGG**  TCTCGCTGAAACTATGCGTTATC | GTTATTTTCTATGACGATGCTTTC |
| GST  antisense strand | GTCTCGCTGAAACTATGCGTTATC | GGATCC**TAATACGACTCACTATAGG**TTATTTTCTATGACGATGCTTTC |
| GFP  sense strand | GGATCC**TAATACGACTCACTATAGG**  AAGTTCAGCGTGTCCG | CACCTTGATGCCGTTC |
| GFP  antisense strand | AAGTTCAGCGTGTCCG | GGATCC**TAATACGACTCACTATAGG**CACCTTGATGCCGTTC |

Note: The T7 promoter sequence is bolded in RNAi


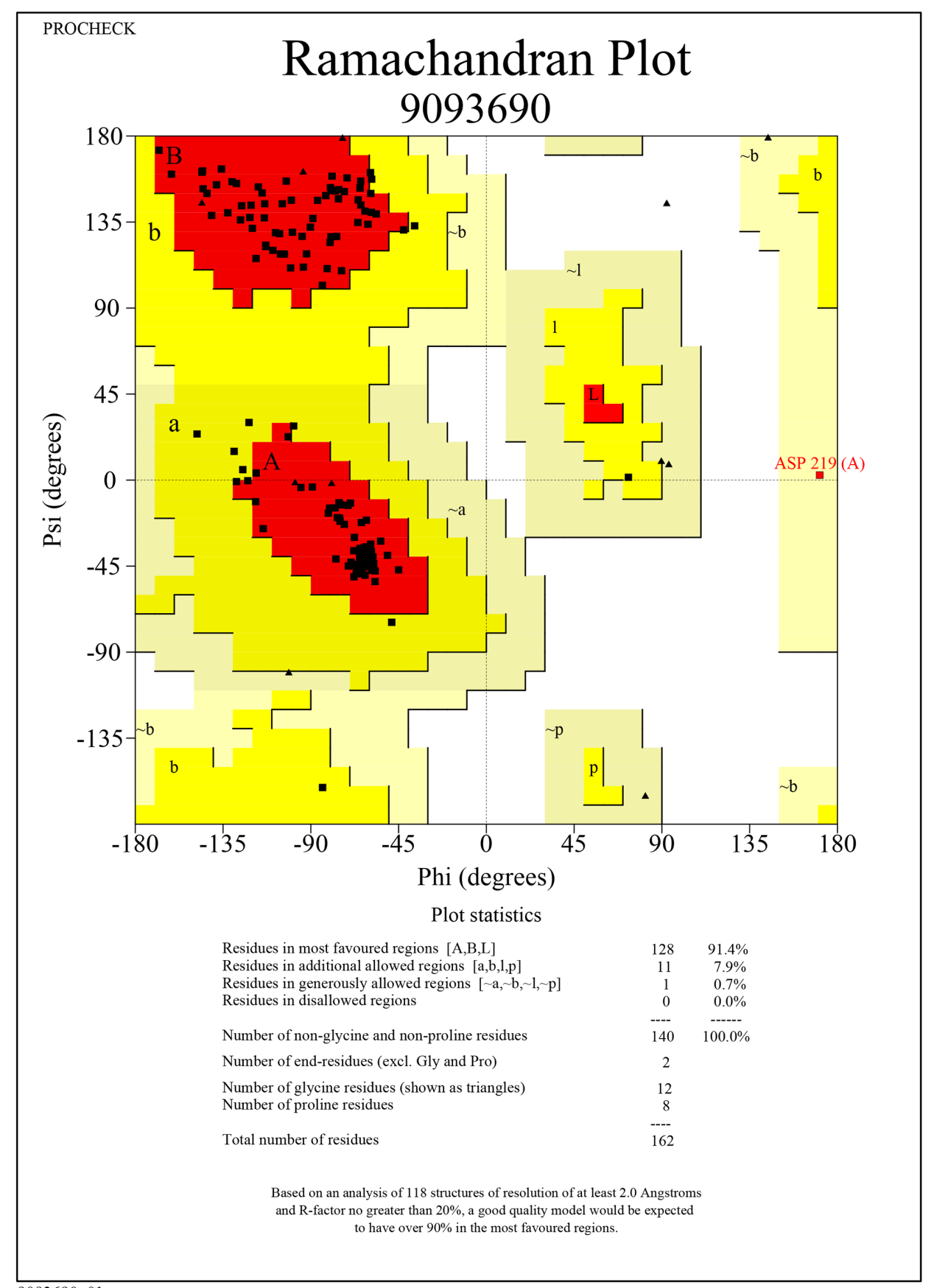


PGRP-SC1

**a**


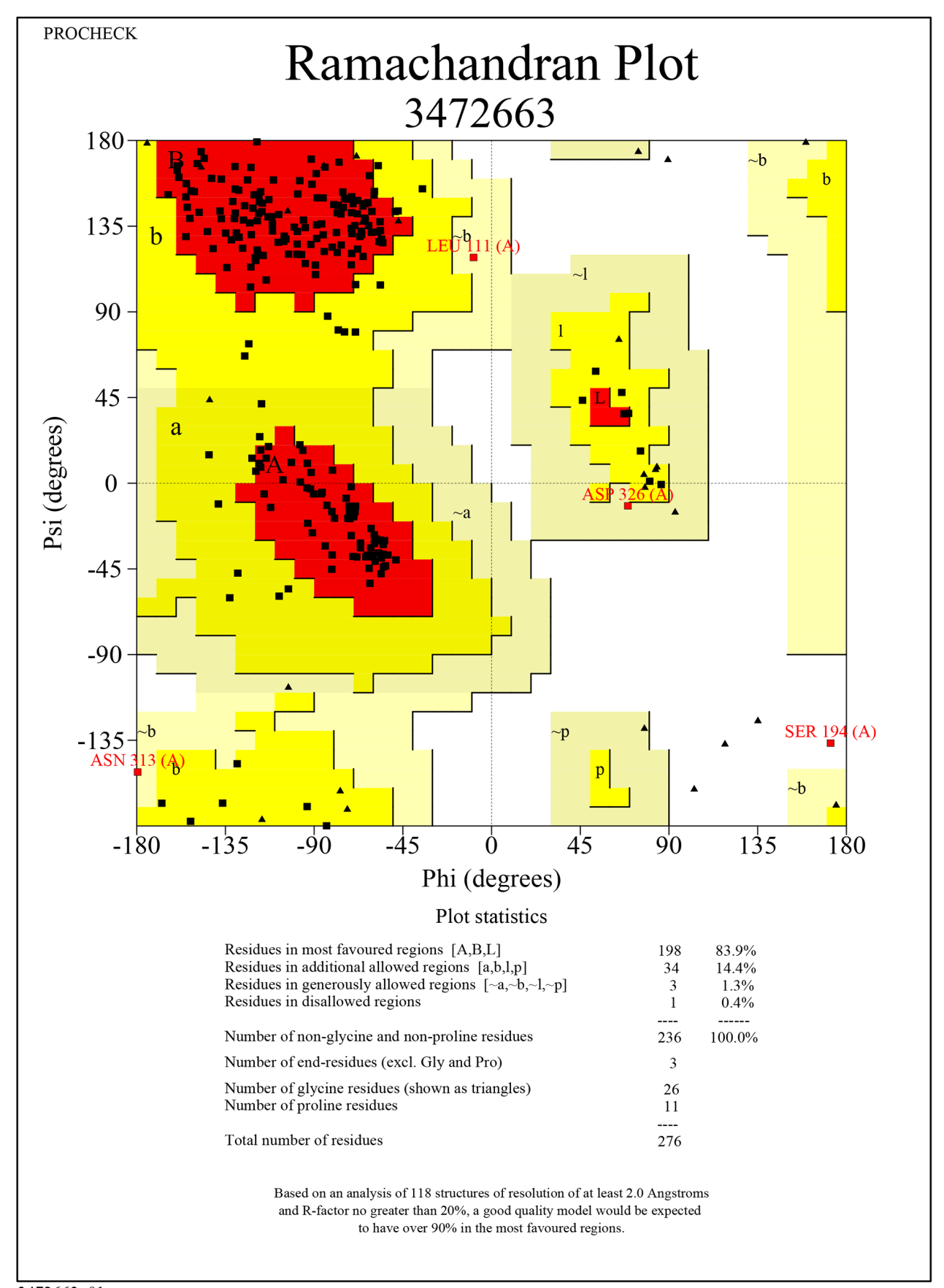


**b**

PPAE-I

**Supplementary Figure 1.** Rationality evaluation results of PGRP-SC1 (a), PPAE-I (b) and GST (c) 3D model. The red regions (A, B, L) represents the residues in most favoured regions; Bright yellow regions (a, b, l, p) represents the residues in additional allowed regions; Dark yellow regions (~a, ~b, ~l, ~p) represents the residues in generously allowed regions


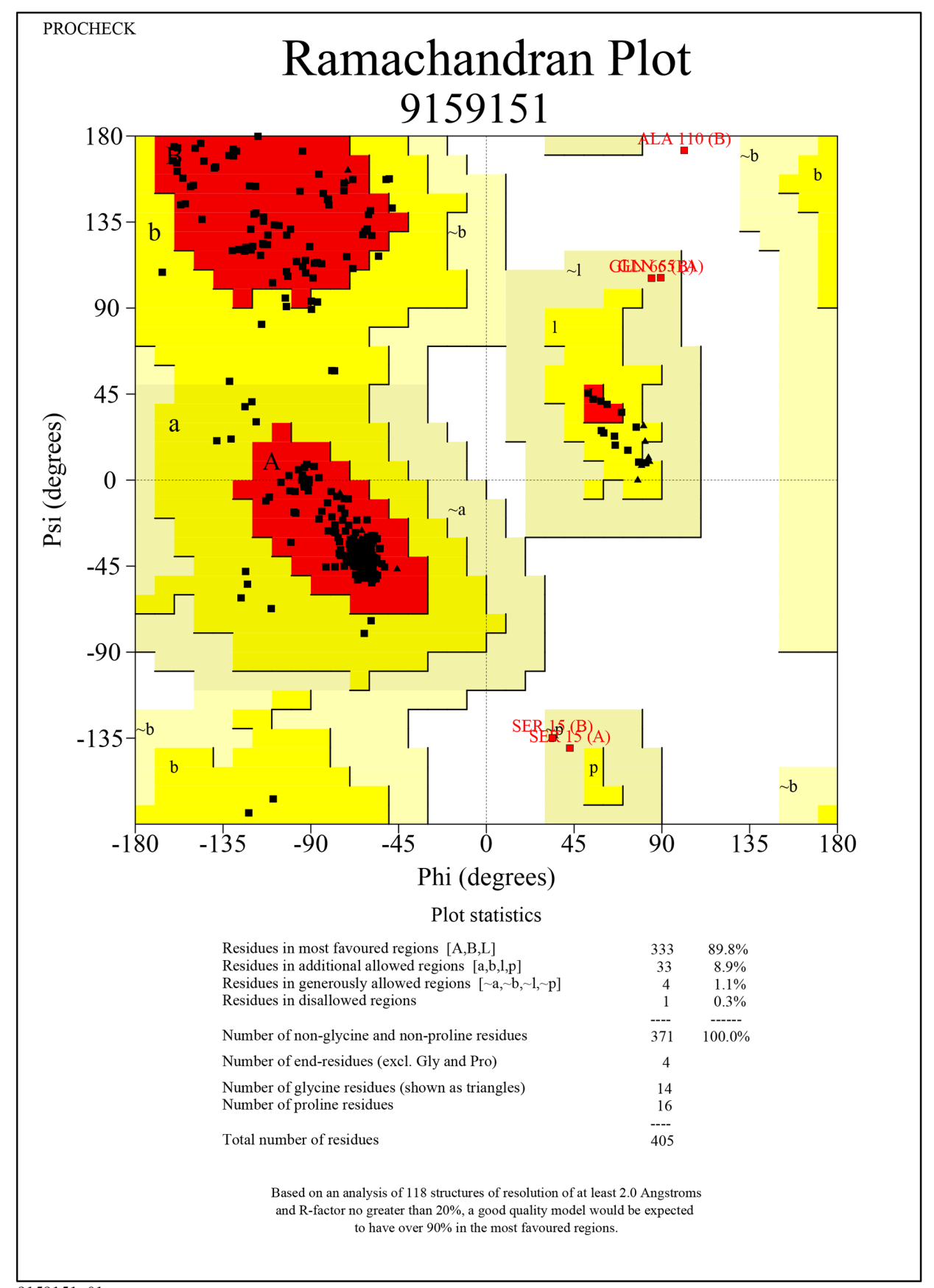


**c**

GST
